# Effect of Age on Flow-Rate, Protein and Electrolyte Composition of Stimulated Whole Saliva in Healthy, Non-Smoking Women 

**DOI:** 10.2174/1874210600802010089

**Published:** 2008-06-11

**Authors:** Liisi Sevón, Merja A Laine, Sára Karjalainen, Anguelina Doroguinskaia, Hans Helenius, Endre Kiss, Marjo Lehtonen-Veromaa

**Affiliations:** 1 Department of Periodontology, Institute of Dentistry, University of Turku, Finland; 2 Department of Paediatric Dentistry, Institute of Dentistry, University of Turku, Finland; 3 Department of Biomedical Sciences, University of Turku, Finland; 4 Department of Periodontology, University of Szeged, Hungary; 5 Public Health Center of Raisio, Finland

**Keywords:** Salivary electrolytes, age, reference values

## Abstract

As relatively little is known about the effect of age on salivary electrolytes we studied the composition of saliva as function of age to provide reference values for healthy non-smoking women. All non-medicated and non-smoking 30-59-year-old subjects (n=255) selected from among 1030 women participating in a screening program formed the material of the present study. Salivary calcium, inorganic phosphate, magnesium, sodium, potassium, protein and flow-rate of stimulated whole saliva were measured. We found age-related changes in salivary calcium and phosphate concentrations (p=0.001 and p=0.004, respectively, one-way ANOVA). Peak values occurred at around 50-54 years of age. Age had no effect on flow-rate, magnesium, sodium, potassium or proteins. The concentration of sodium correlated positively, while phosphate, potassium, magnesium and protein correlated negatively with the salivary flow-rate. Calcium was the only electrolyte which had no association with flow-rate. Our study provides reference values for salivary electrolytes of 30-59-year-old women.

## INTRODUCTION

Salivary research is an important field of dentistry and oral biology. The significance of flow-rate and pH of saliva in the development of caries have been well-established already in the late 1970s and early 1980s [1]. Sex-dependent differences in flow rate [2] and calcium content of saliva [3, 4] have been observed. Clinically these findings suggest connections with caries on one hand and with chronic gingivitis and periodontitis on the other [4-7]. Apart from calcium there is little information about the association between salivary electrolytes and oral health. The significance of other electrolytes is unclear but it has been shown that medications can affect the composition of salivary electrolytes [8]. Moreover, the available textbook information on the concentration of inorganic components of whole saliva is mixed due to varying collection techniques [9]. Information on smoking or medications, factors known to affect the composition of saliva, are rarely given in the original publications cited in the review of Ferguson [9].

Earlier studies indicate that salivary calcium content increases with increasing flow-rate, as stimulation increases the calcium level of submandibular saliva [10, 11]. Yet, our follow-up study on stimulated whole saliva of menopausal women demonstrated that salivary flow-rate and calcium content are not directly correlated [12]. It is known that estrogens affect oral health in a number of ways, and saliva undergoes variations during e.g. pregnancy and menopause [13, 14]. Dry mouth is a common complaint among older women. Aging process, however, is not the primary cause of reduced salivary flow rate [2], but secondary to various diseases and/or medications [2]. Therefore reference values for organic and inorganic composition of saliva are needed. There are only few age-related salivary studies on non-medicated subjects, but to our knowledge no salivary studies exist excluding both the effects of medications and smoking.

Our aim was to study the effect of age on salivary flow rate, the level of calcium, phosphate, magnesium, sodium and potassium in healthy women. These results can be used as reference values for 30-59-year-old women.

## MATERIALS AND METHODS

Originally our study group consisted of 1030 women (age range 30-62 years) participating in a pre-screen referral program for osteoporosis. The screening was carried out by the Public Health Centre of Raisio, a South-Western Finnish community with a population of 23 000 inhabitants. The age cohorts invited in 1999 included all women living in the community and born in the years 1940, 1941, 1943, 1945, 1949, 1954, 1957, 1959, 1964 and 1969. There was one subject born in 1937 who participated in the screening but was excluded from the present study. Women with verified (n=12) and uncertain pregnancies (n=3) were excluded. A brief medical history including medications and smoking habits were recorded by a questionnaire filled out by all consenting participants before screening. All participants having one or several systemic diseases or using medications including hormone replacement therapy were excluded. Women who reported of smoking habits were also excluded. The age distribution of the remaining healthy, non-medicated, non-smoking subjects (n=255, 30-59 years) is presented in Table **1**. The women were further divided in subgroups at five-year intervals.

The study was approved by the ethics committee of the municipality of Raisio. The subjects were volunteers and informed consent was obtained from all participants.

The participants refrained from tooth brushing, eating, and drinking for a minimum of one hour prior to saliva collection. The collection procedure of the saliva samples was standardized prior the study. The samples were collected around noon in field conditions without any laboratory equipments at the collection site. Stimulated whole saliva was collected by chewing a piece of paraffin-wax (1 g) at habitual pace. After 60 s of pre-stimulation, the secreted saliva was spat in graded disposable plastic cups for 5 minutes. The flow rate was measured and expressed as ml/min. The samples were transferred to test tubes immediately after collection, put on ice, frozen and stored at –20(C until further analysis.

Calcium, magnesium, potassium and sodium concentrations were measured by atomic absorption spectrophotometer (Perkin-Elmer Atomic Absorption Spectrophotometer Model 303, Norwalk, USA). Due to the strong affinity of calcium to form complexes with salivary proteins, non-centrifuged whole saliva containing both protein-bound and soluble calcium was used for the assay. All other analytical procedures were carried out using centrifuged (12 000 *g*, 10 min +4º C) and diluted samples as recommended by the manufacturer [15]. Inorganic phosphate was analyzed according to Kallner [16] and total protein according to Lowry *et al*. [17], both from centrifuged saliva.

The normality of distributions of the response variables were controlled by the Kolmogorov-Smirnov test. Before statistical analyses, logarithmic transformations of the salivary variables were made due to the skewed distributions. The statistical evaluations were performed by one-way analysis of variance. Correlations between flow rate and salivary constituents were measured by Pearson's correlation coefficients. A commercial software program (Statistical Package for Social Sciences for Windows, version 9.0, SPSS Inc., Chicago, Illinois, USA) was used to run the statistical analyses.

## RESULTS

The average flow rates of paraffin-stimulated saliva in the six age groups varied from 1.4 (±0.6) to 1.8 (±0.7) ml min, yielding an average of 1.5 (±0.7) ml/min for the whole study group (Table **1**). The average flow rate of the second age group (35-39) was slightly higher than the rest of the age groups but the difference was not significant (p=0.182).

Salivary calcium and phosphate concentrations showed a clear increase with increasing age (Table**2**). Peak values occurred at around 50-54 years of age. Calcium and phosphate increased about 12 % at the age group of 50-54 years as compared to the younger age group from 45 to 49 years.

Salivary flow rate correlated negatively with magnesium, potassium, and phosphate, and with the protein level and positively with sodium. Calcium was the only electrolyte which did not show correlation with flow rate (Table **3**).

Means and standard deviations (SD) of salivary flow rate, sodium, potassium, magnesium concentrations and protein content and 97.5% and 2.5% percentiles of all age groups are given in Table**4**.

## DISCUSSION

To our knowledge this is the first time when salivary composition has been studied for reference purposes in non-medicated and non-smoking women to this extent. We are providing values for randomly selected subjects, contrary to many earlier studies. The main difficulties in salivary research of adult and elderly population are the great inter-individual variation, the increasing number of subjects using medications and having diseases affecting salivary flow and composition. Only 22% of the initial study group of 1030 women met our strict criteria. Flow-rate correlated positively with sodium and negatively with phosphate, potassium, magnesium and protein, which is partly in line with the most recent text-book data [9]. However, some of our findings are controversial as compared to earlier reports: we found that salivary potassium was negatively correlated with flow rate as opposed to earlier reports [18] showing that potassium after a 2-3 minutes' continued stimulation reached a constant value. This may be due to two reasons: firstly, we studied whole saliva while the results of the study above [18] apply to parotid saliva, and secondly we made inter-individual comparisons as opposed to the study-design above [18] where intra-individual variations are presented as function of prolonged stimulation. Calcium was the only electrolyte in our study, which did not correlate with salivary flow-rate. This is in contrast to earlier studies showing an increase in salivary calcium with short-term citric acid stimulation of parotid saliva [11].

According to our study, salivary calcium and phosphate concentrations increase with age showing peak values around menopause. Therefore we suggest that menopause is reflected in saliva as elevated levels of calcium and phosphate. This result is well in accordance with our earlier findings [12]. The reason why salivary calcium seems to increase with age may be explained by the hypothesis we have presented earlier for smokers: we were suggesting that a decrease in skeletal bone density, frequently detected in elderly people, may increase the amount of calcium in saliva [19]. However, this phenomenon is not completely clear and needs further studies. We have data on salivary calcium of different study populations with decreasing bone mineral density, such as patients with rheumatoid arthritis [20], heavy smokers [19] and women in menopausal ages [12]. They all have higher means of salivary calcium level when compared to age-matched counterparts. We have also found that hormone replacement therapy, which has a stabilizing effect on calcium content of bone, has a similar effect on salivary calcium [12].

Earlier it was generally believed that salivary flow rate decreases with age, but increasing number of studies are showing that aging does not affect the rate of stimulated whole saliva. Our current finding of no correlation between age and salivary flow-rate is well in line with the works of Parvinen and Larmas [2], Tylenda *et al*. [21], and with the more recent studies of Närhi *et al*. [22], Percival *et al*. [23], and Yeh *et al*. [24].

To conclude, we found that age had no effect on the flow-rate of stimulated saliva. However, salivary calcium and phosphate concentrations increased with age showing peak values around 50 years of age. In addition, normal reference values of salivary electrolytes of different age groups are provided for women to enable future diagnostic use of salivary electrolytes.

## Figures and Tables

**Table 1.** Frequency Distribution and Salivary Flow Rate of Healthy, Non-Medicated and Non-Smoking Women According to Age Groups. The p-Value for Variations in Flow Rate was 0.128 (One-way ANOVA)

Age Groups	N	Flow Rate ml/min Mean (SD) (Min-Max)
30-34	23	1.4 (0.6) (0.5-3.0)
35-39	28	1.8 (0.7) (0.8-3.4)
40-44	44	1.4 ( 0.6) (0.6-3.2)
45-49	54	1.4 (0.6) (0.4-3.1)
50-54	69	1.6 (0.8) (0.3-4.0)
55-59	37	1.6 (0.7) (0.4-3.2)
Total	255	1.5 (0.7) (0.3-4.0)

**Table 2.** Salivary Calcium and Phosphate in 30-59-Year-Old Non-Medicated and Non-Smoking Women. The Age-Related p-Values for Calcium and Phosphate were 0.001and 0.004, Respectively (One-way ANOVA)

Age Group	Calcium (mmol/l) Mean (SD)	2.5 Percentile	97.5 Percentile	Phosphate (mmol/l) Mean (SD)	2.5 Percentile	97.5 Percentile
30-34	1.24 (0.26)	0.82	1.82	3.77 (1.00)	2.38	6.32
35-39	1.16 (0.23)	0.87	1.71	3.37 (1.00)	1.43	5.1
40-44	1.40 (0.37)	0.73	2.15	3.82 (0.89)	2.27	5.82
45-49	1.43 (0.28)	0.94	1.9	3.71 (1.14)	1.9	6.32
50-54	1.73 (0.41)	1.2	2.52	4.38 (1.57)	2.32	8.95
55-59	1.61 (0.29)	1.15	2.42	4.04 (1.27)	1.57	8.47

**Table 3.** Correlations between Salivary Flow Rate and Cal-cium, Phosphate, Magnesium, Sodium, Potassium and Protein

	Pearson’s Correlation (p-Value)	Regression Coefficient
Calcium (mmol/l)	0.02 (0.750)	0.071
Phosphate (mmol/l)	-0.33 (0.000)	-3.764
Magnesium (mmol/l)	-0.34 (0.000)	-0.144
Sodium (mmol/l)	0.40 (0.000)	17.85
Potassium (mmol/l)	-0.31 (0.000)	-10.07
Proteins (g/l)	-0.25 (0.000)	-0.025

**Table 4.** Means, Standard Deviations and 2.5 and 97.5 Percentiles of Salivary Flow Rate, Sodium, Potassium, Magnesium and Protein of 255 Non-Smoking and Non-Medicated Subjects

	Mean (SD)	2.5 Percentile	97.5 Percentile
Flow rate (ml/min)	1.5 (0.7)	0.4	3.2
Sodium (mmol/l)	10.8 (6.7)	2.8	27.1
Potassium (mmol/l)	18.5 (3.4)	10.9	25.5
Magnesium (mmol/l)	0.104 (0.060)	0.039	0.221
Protein (g/l)	1.21 (0.34)	0.70	2.06
